# Molecular and immunologic markers of kidney cancer—potential applications in predictive, preventive and personalized medicine

**DOI:** 10.1186/s13167-015-0042-2

**Published:** 2015-10-20

**Authors:** Amanda Mickley, Olga Kovaleva, Julia Kzhyshkowska, Alexei Gratchev

**Affiliations:** Institute of Transfusion Medicine and Immunology, Medical Faculty Mannheim, Ruprecht-Karls University of Heidelberg, Mannheim, Germany; Blokhin Cancer Research Center, Moscow, Russia; Red Cross Blood Service Baden-Württemberg—Hessen, Mannheim, Germany; Laboratory for Translational Cellular and Molecular Biomedicine, Tomsk State University, Tomsk, Russia; Laboratory of the Tumour Stromal Cells Biology, Institute of Carcinogenesis, Blokhin Cancer Research Center, Kashirskoye Shosse 24, Moscow, Russia

**Keywords:** Kidney, Renal cell carcinoma, Mutation, Epigenetics, Tumor-associated macrophage, Predictive, preventive and personalized medicine, Biomarker panel, Metastatic disease, Molecular target, Immune response, Disease prognosis

## Abstract

Kidney cancer is one of the deadliest malignancies due to frequent late diagnosis (33 % or renal cell carcinoma are metastatic at diagnosis) and poor treatment options. There are two major subtypes of kidney cancer: renal cell carcinoma (RCC) and renal pelvis carcinoma. The risk factors for RCC, accounting for more than 90 % of all kidney cancers, are smoking, obesity, hypertension, misuse of pain medication, and some genetic diseases. The most common molecular markers of kidney cancer include mutations and epigenetic inactivation of von Hippel-Lindau (VHL) gene, genes of vascular endothelial growth factor (VEGF) pathway, and carbonic anhydrase IX (CIAX). The role of epigenetic pathways, including DNA methylation and chromatin structure remodeling, was also demonstrated. Immunologic properties of RCC enable this type of tumor to escape immune response effectively. An important role in this process is played by tumor-associated macrophages that demonstrate mixed M1/M2 phenotype. In this review, we discuss molecular and cellular aspects for RCC development and current state of knowledge allowing personalized approaches for diagnostics and prognostic prediction of this disease. A set of macrophage markers is suggested for the analysis of the association of macrophage phenotype and disease prognosis.

## Introduction

Kidney cancer is one of the most common and deadly malignancies, with approximately 58,000 new cases and 13,000 deaths estimated to have occurred in the USA in 2010 (http://www.cancer.gov/cancertopics/types/kidney). There are two main types of kidney cancer, which includes renal cell carcinoma (RCC) and renal pelvis carcinoma. RCC is the most prevalent, accounting for 90 % of all kidney cancers with 33 % of these cases being metastatic at diagnosis [1]. Risk factors for the development of RCC include cigarette smoking, obesity, continued misuse of pain medications, acquired cystic kidney disease, hypertension, and other genetic diseases [2, 3]. The major problems in treating RCC are late diagnosis and poor response to available therapies.

RCC arises from the proximal renal tubular epithelium and involves several different cell types, such as clear cells, granular cells, and spindle cells. The proximal renal tubule plays an important role in homeostasis, regulating pH, salt concentration, glucose concentration, and other substances by reabsorption into the blood or excretion with the urine [4]. There are both genetic and epigenetic alterations that are connected to RCC development. Genetic disorders leading to RCC and their genes of mutation can be seen in Table 1 [5]. The most prevalent genetic cause of RCC is von Hippel-Lindau syndrome, a disorder characterized by the development of vascular tumors such as RCC, hemangioblastomas of the central nervous system and pheochromocytoma [5, 6]. The von Hippel-Lindau (VHL) gene on chromosome 3 (3p25–26) has been identified as a tumor suppressor gene for this syndrome [5]. The VHL gene is inactivated in 75 % of RCC cases [7]. Patients inherit one mutated allele and acquire a mutation in the second allele of the affected organs. Patients who have non-inherited Hippel-Lindau syndrome have acquired a mutation in both alleles [5].

**Table 1:** Hereditary renal cell syndromes with genes of mutation

Syndrome	Gene (chromosome)
von Hippel-Lindau	VHL (3p25–26)
Hereditary papillary renal carcinoma	C-Met proto-oncogene (7q31–34)
Hereditary leiomyomatosis RCC	Fumarate hydratase (1q42–43)
Birt–Hogg–Dubé	BHD1 (17p11)
Tuberous sclerosis	TSC1 (9q34) or TSC2 (16p13)

Modified from [5]

### Molecular predictive and prognostic markers

The three main predictive and prognostic markers used for RCC are VHL, vascular endothelial growth factor (VEGF), and carbonic anhydrase IX (CAIX) [3, 8]. The first of which, VHL, has been studied immensely as a biomarker. In a study by Yao et al., it was shown that the presence of VHL alterations is associated with better outcomes for patients with stages I–III clear cell RCC treated by nephrectomy [9]. Mutational analyses showed that almost 100 % of related RCC patients have germ line VHL alterations; 42–57 % of sporadic clear cell RCC patients have somatic intragenic mutations, and in 5–19 % of these tumors, aberrant hypermethylation of VHL has also been found [9]. In another study by Choueiri et al., it was found that patients with an alteration of the VHL gene had a better response to anti-VEGF therapy as opposed to those with the wild-type gene [10].

VEGF is an important ligand involved in tumor angiogenesis. When patients are effectively treated with VEGF-targeted therapy, VEGF levels in the serum rise and soluble VEGFR levels decrease. Therefore, these factors are used as pharmacodynamic biomarkers [3]. In a study by Rini et al., serum levels of these factors were measured before and during treatment with VEGF-targeted therapy. Patients with baseline levels of sVEGFR-3 were associated with a better prognosis, suggesting that these factors can be used as predictive markers in this type of therapy [11]. Also, VEGF is a downstream marker of HIF activation, making it a marker for VHL loss and HIF deregulation [3].

CIAX is a protein induced by hypoxia, which is widely found in RCC cells. This protein regulates the conversion of carbon dioxide to carbonic acid, leading to pH homeostasis in tumors during hypoxic conditions and the accumulation of lactic acid [3]. CIAX has proved to be an effective prognostic marker. In a study by Sandlund et al., RCC patients were evaluated using tissue microarray analysis and CAIX staining; patients with more than 91 % of staining were associated with a better prognosis than patients with low amounts of staining [12, 13]. In another study by Li et al., serum levels of CAIX were analyzed in patients; the level of CAIX was statistically significant in relation to tumor stage, grade, and size [14]. CAIX seems to be a sound marker for prognostic purposes and for VHL/HIF deregulation.

There are further molecules that can be considered as diagnostic and prognostic markers of kidney tumors. These include HIF-1, Survivin, mTOR, CA9, PTEN, tyrosine kinases Akt and S6K, cytokines CCL5 and CXCL9, caveolin-1, and some others. However, most of these proteins are not specific for kidney pathology. For example, Survivin, belonging to the family of apoptosis inhibitors, can be considered as a ubiquitous marker of various cancers, since its expression cannot be detected in differentiated cells of an adult organism and remains detectable only in dividing cells. Quantitative analysis of its expression can also be used as a marker of poor prognosis [15]. Inactivation of PTEN phosphatase is as well typical for the majority of tumor types—glioma, meningioma, melanoma, kidney tumor, hepatoma, cervical, mammary, and prostate carcinoma, so that this marker cannot be considered specific too. Tyrosinkinases Akt and S6K are the components of mTOR signaling pathway, regulating initiation of protein translation in the cell, being responsible for general cell metabolism. Literature data indicate that analysis of рAkt and рS6K expression level in 20 patients undergoing temsirolimus therapy can be used for prediction of the efficacy of mTOR-targeting therapy [16]. Expression of caveolin-1, the component of membrane caveole, is associated with bad prognosis of prostate, lung, mammary gland, kidney, and esophagus tumors [17]. Protooncogene Bcl-2 is a candidate marker of a good prognosis in kidney cancer. In a study of 101 cases of kidney tumors, a correlation with good prognosis of disease was observed [18]. However, there are other studies, indicating that there is no correlation between Bcl-2 and prognosis of the disease [19, 20]. More specific seems to be the analysis of several markers. For instance, simultaneous increase of caveolin and the components of Akt/mTOR-mediated signaling is a strong indicator of poor prognosis of kidney cancer [21].

In addition to mutations, epigenetic gene regulation by modifying DNA methylation is a further major mechanism of cancer initiation and progression. There is clear evidence that DNA hypermethylation of CpG islands around the promoter regions silences tumor-suppressor genes [22]. This process in turn closely couples with and depends on histone deacetylation. In fact, abnormal histone acetylation status has been demonstrated to initiate undesirable phenotypic changes and cancer [23]. Hence, histone deacetylases (HDAC) inhibitors are supposed to be useful in cancer prevention, due to their ability to “reactivate” the expression of epigenetically silenced genes, including those involved in differentiation, invasion, and metastasis. During the last years, it was shown that HDAC blockade significantly reduces RCC growth and invasion in vitro and in vivo [24–26]. Nevertheless, downregulating HDAC alone seems not to be sufficient to induce long-lasting antitumor effects in clinical trials. It is therefore necessary to further optimize the treatment protocol, either by introducing an additional compound [27, 28] or by identifying very specific target structures, manipulation of which strongly stops progressive RCC dissemination.

### Immune escape

It is believed that RCC is partially controlled by the immune system because of the frequency of the disease in immunosuppressed people, the amount of T cell infiltration to the tumor sites, and the ability of metastasis in these individuals to regress spontaneously. Immune escape in these tumors is caused by alterations in immune cells, the tumor microenvironment, tumor loss of function, and tumor gain of function [29].

Around 40 % of RCC lesions have at least a partial loss of MHC class I antigens, and 6 % show a complete loss [30]. MHC class I antigen processing and presentation is important for the recognition of tumor cells by CD8+ cytotoxic T lymphocytes (CTLs). Other molecules involved in antigen presentation, such as the transporter-associated with antigen processing (TAP) subunit 1, the chaperone tapasin, and low-molecular weight protein 2 and 7 (LMP2 and LMP7) are also regularly downregulated in RCC cells in comparison to surrounding normal kidney epithelium [29]. It has also been found that the RCC cells lack costimulatory molecules B7-1 and B7-2. The lack of these cells causes deficient T cell activation leading to T cell anergy and apoptosis.

RCC cells also escape immunity due to gains in function. HLA-G has been found to be upregulated in RCC. This protein is involved in the immune system by interacting with immunoglobulin-like killer inhibitory receptors (KIRs) that are found on natural killer (NK) cells and T lymphocytes [29]. When HLA-G is bound to these receptors, it protects tumor cells from attack from these cells. Effector cells can also be killed due to an increase in apoptosis-inducing molecules. It was found that when T cells were cocultivated with RCC cells, more T-cells went into apoptosis than normal. This may be because of an upregulation of tumor necrosis factor (TNF)-related apoptosis inducing ligand (TRAIL), Fas ligand (FasL), or CD70 [31].

Alterations in T cells and other immune cells also lead to a decreased immune response in RCC patients. DCs are poorly recruited to tumor tissues, and T cells are silenced by the secretion of factors involved in the TCR signaling transduction pathways. A downregulation of the TCR zeta chain and p56 (Ick) has been found in the tumor infiltrating lymphocytes of these patients, impairing T cell activation [29].

The tumor microenvironment plays highly important role in the RCC cells’ ability to escape immune responses. The infiltration of lymphocytes, tumor-associated macrophages, and DCs to the tumor sites contributes to the tumor. These cells are able to produce factors such as TNF-α or IL-6 which promote tumor growth and IFN, IL-4, or IL-13 that prevent proliferation [29, 31].

### Tumor-associated macrophages in kidney cancer

The tumor is comprised of a heterogeneous microenvironment of both malignant and normal stromal cells [32] and contains a large amount of macrophages called tumor-associated macrophages (TAMs). These macrophages have the ability to reduce tumor growth via non-specific cytotoxic mechanisms or specific cell lysis [33, 34]. However, in most of the cases, TAMs are modified in a way to aid in malignant cell progression, invasiveness, and evasion of apoptosis [32]. Chemokines such as monocyte chemotactic protein-1 (MCP-1), macrophage colony stimulating factor (M-CSF), and VEGF are secreted by the tumor cells and attract macrophages to the site of malignancy [35].

A variety of traits possessed by macrophages can then aid in the progression of the tumor. One example is the macrophage’s ability to aid in angiogenesis through the release of angiogenic factors, such as cytokines and matrix metalloproteinases. Tumors need angiogenesis in order to grow and for metastasis. The process of angiogenesis includes extracellular matrix degradation, proliferation and migration of capillary endothelial cells and differentiation of these cells into mature capillaries, providing a route for nutrients and oxygen to enter the tumor and for metastatic cells to exit the tumor into circulation [35].

Tumor-associated macrophages have also been shown to aid in tumor growth. These macrophages secrete various factors such as EGF, TGF-β1, and basic FGF when cocultured with tumor cells aiding in proliferation and survival. Tumor-associated macrophages have also been shown to be essential for tumor development in macrophage depletion studies [36].

It has been shown that macrophages exposed to apoptotic tumor cells have reduced cytotoxicity and nitric oxide production in response to interferon-gamma and lipopolysaccharide. It has also been shown that macrophages exposed to apoptotic tumor cells increase the growth of tumor cells by 40 % and tumor-associated macrophages promote tumor angiogenesis, invasion, intravasation, and metastasis in animal models [37].

In kidney tumors, an association between the amount of TAMs is well established. It was demonstrated that high infiltration of tumor with CD163+ cells correlates with poor clinical prognosis [38]. In the same study, the authors demonstrate that direct coculture of macrophages with RCC cells induces type 2 macrophage phenotype. This is explained by the expression of membrane-type M-CSF on the surface of RCC cells. At the same time, it was shown that TAMs isolated out of RCC express not only immunosuppressive IL-10 but also pro-inflammatory chemokine CCL2 [39]. These macrophages also showed enhanced eicosanoid production via activated 15-lipolxygenase-2 pathway [39], what is typical for activation of macrophages by TGFβ [40]. Though unexpected, another specific property of TAMs in RCC is expression of CCR8 associated with higher activity of Stat3-mediated signaling, what is rather typical for inflammatory phenotype. These cells are considered to be capable of stimulating FoxP3 expression in T cells and have proangiogenic activity [41]. There are more confirmations of pro-inflammatory phenotype of TAMs in RCC. Petrella and Vincenti reported that increased expression of IL1β, a cytokine that may be produced by inflammatory macrophages, contribute to aggressive RCCs. This cytokine leads to expression of matrix metalloproteinases (MMPs)−1, −3, and −10 that contribute to tumor cell invasion [42].

There is also a connection between TAM density and VEGF pathway. In the study by Toge et al., it was found that in more progressive RCC, the VEGF level was higher what correlated with higher numbers of TAM and higher microvessel density. Also, VEGF level and TAM were significantly higher in patients with recurrence. The authors identified VEGF and TAM numbers as prognostic factors. Moreover, TAM was the only independent prognostic factor by multivariate analysis [43]. These data are supported by the study, demonstrating that VEGFR1 knockdown leads to reduced macrophage infiltration in the tumor [44].

It is clear that RCC TAMs show mixed phenotype what makes the analysis of the balance between M1 and M2 type activation even more important. In the paper of Xu et al., it was demonstrated that the number of TAMs has only limited prognostic value while the analysis of CD11c/CD206 balance provides a good possibility to determine individual prognosis (low CD11c and high CD206 densities are associated with poor prognosis) [45].

Although most of the studies are performed on clear cell RCC, the importance of TAMs was demonstrated also for papillary renal cell carcinoma. Papillary RCC can be subdivided in subtypes I and II based on histological criteria. Type II is associated with poor prognosis. Although density of TAM CD68+ tam was similar in both subtypes, the analysis of more specific markers revealed that nearly all macrophages in type II tumors express CD163, while in type I tumors, there were less than 30 % CD163 positive. Higher number of CD163 cells was associated with higher density of capillaries as defined by CD31 staining. These findings suggest the functional impact of TAMs on the prognosis of papillary RCC type II [46].

### Personalized therapy

Since the loss of VHL expression (50–80 % cases of clear cell renal cell carcinoma) is the most frequent molecular event observed in kidney tumors, most of the targeted drugs used for the treatment of this pathology are inhibitors of various components of signaling pathways related to VHL inactivation. However, it is important to note that kidney tumors other than renal cell carcinoma, i.e., papillary, chromophobe, collective duct, and other types of renal cell carcinoma are not associated with VHL inactivation. Therefore, search for new molecular markers and signaling pathways which components can become therapeutic targets is necessary for effective treatment of RCC.

The currently available therapeutic agents for advanced RCC include one monoclonal antibody (bevacizumab), four TKIs targeting VEGF receptors (VEGFRs) (sorafenib, sunitinib, pazopanib, and axitinib), two inhibitors of the mammalian target of rapamycin-related complexes (temsirolimus and everolimus), and one recombinant form of an endogenous cytokine (interleukin-2). At present, sunitinib and pazopanib are the most frequently used first-line treatments for advanced RCC. However, 25 % of patients receiving this treatment will not obtain any benefit, demonstrating early progression of the disease. Secondary, resistance is also an important problem, as most patients who initially respond to these agents will eventually progress [47].

To increase RCC therapy efficacy, targeted drugs are combined with antiangiogenic ones. The latter include drugs that block HIF1α-VEGF signaling at several levels, for example, combination of drugs that block VEGF and its receptors. Other type of combined drug includes blocking of several signaling pathways simultaneously, i.e., VEGF, PDGF, EGF signaling pathways and mTOR pathway [48, 49].

Several germline studies have investigated the effect of polymorphisms on the efficacy and toxicity of antiangiogenic agents, mainly focusing on genes-encoding enzymes involved in metabolism (CYP3A4, CYP3A5, and their regulator NR1I3), transporters (ABCB1 and ABCG2), and targets (VEGF, VEGFRs, and FLT3) of these drugs. The first pharmacogenetic study of sunitinib supported the impact of SNPs in *NR1I3*, *ABCB1*, and *CYP3A5* on progression-free survival [50], and a subsequent study revealed an association between two *VEGFR3* missense polymorphisms and poor outcome with sunitinib treatment [51].

In a recent study of patients treated with pazopanib, Xu et al*.* [52] found an association between progression-free survival and SNPs in *IL8* and *HIF1A*, and the association between *UGT1A1* polymorphisms and bilirubin elevation in pazopanib-treated patients has recently been confirmed. Also, higher responses to antiangiogenic drugs were reported in familial VHL-RCC (i.e., RCC with germline *VHL* mutations) in some but not all studies [53–55].

Despite active development of therapeutic approaches for RCC, it remains unclear how to select the optimal therapy for a particular patient. In contrast to adenocarcinoma of the lung or colon where mutations in *EGFR* or *K-RAS* are the markers clearly predicting the sensitivity of tumor cells to EGFR inhibitors, the presence of VHL mutations is not a clear indication for the use of VEGF/VEGFR inhibitors or other antiangiogenic drugs. It is not yet clear which VHL mutations lead to a complete loss of function [56, 57].

Therefore to date, the clinical benefit an individual patient will derive from antiangiogenic therapy is highly variable and largely unpredictable. Between 20 and 30 % of patients with ccRCC derive no benefit from first-line TKI treatment [47, 58]. In addition, these drugs are toxic and expensive. There is thus great impetus to discover biomarkers in RCC that can identify the subpopulation of patients destined to gain maximal benefit from any given drug.

### Expert recommendation

Molecular markers, identified during last years provide an excellent possibility for the development of a personalized and predictive approach for patient stratification and development of treatment strategy. Classification of these markers according to the recommendations of EPMA provided in the section “Biomarker Discovery, Validation, Standardisation and Practical Application in Medical Practice” of the EPMA White Paper [59] will improve the usage of these markers in clinical practice.

Data is available for genetic and epigenetic changes in the tumor cells and immunologic properties of the tumor. A complex study that will include investigation of all these parameters, i.e., mutations, gene silencing by DNA methylation, and properties of TAMs is needed. Special attention has to be given to tumor-associated macrophages. Research papers indicate that simple measurement of TAMs amount provides insufficient information; therefore, additional research is needed to collect more information about favorable and detrimental macrophage phenotypes. It is important not to limit the analysis to simple discrimination between M1 and M2 macrophage phenotype but to identify the effects of individual cytokines in connection with personal properties of the immune system. This can be achieved using the following markers: CD68, for general macrophage identification; CD163, mannose receptor; Stab1, for general M2 identification; fibronectin, βIG-H3, MMP2, and MMP12, for IL-4-induced effects; Id3, OLR1, HAMP, and YKL-39, for TGFβ-induced effects; MCP1, MCP2, and TNF, for M1 identification.

The early diagnosis of RCC, however, remains to be illusive. There are no reliable soluble markers that can be detected in the blood or urine so far. Therefore, more complex strategies have to be developed for early detection or even prediction of RCC in accordance with the principles of preventive and personalized medicine (PPPM) [60]. These may include analysis of mutation and methylation of genes involved in RCC pathogenesis in circulating tumor cells or in plasma DNA.
